# Alternative Solvents for the Biorefinery of Spirulina: Impact of Pretreatment on Free Fatty Acids with High Added Value

**DOI:** 10.3390/md20100600

**Published:** 2022-09-25

**Authors:** Laura Wils, Mervé Yagmur, Myriam Phelippe, Bénédicte Montigny, Barbara Clément-Larosière, Johan Jacquemin, Leslie Boudesocque-Delaye

**Affiliations:** 1 EA 7502 SIMBA, Faculté de Pharmacie, Université de Tours, 31 Avenue Monge, 37200 Tours, France; 2 Aqua Eco Culture, 7 Rue d’Armor Maroué, 22400 Lamballe, France; 3 EA 6299 PCM2E, Faculté des Sciences et Techniques, Université de Tours, Bât J, Avenue Monge, 37200 Tours, France; 4 MSN Department Mohammed VI Polytechnic University, Lot 660, Hay Moulay Rachid, Ben Guerir 43150, Morocco

**Keywords:** Spirulina, biorefinery, alternative solvents, free fatty acids, pretreatment, deep eutectic solvent

## Abstract

The growing demand for molecules of interest from microalgal biomass, such as phycobiliproteins, has led to an accumulation of unused by-products. For example, phycocyanin, obtained by the extraction of Spirulina, generated cakes rich in non-polar molecules of interest, such as free fatty acids (FFAs). These FFAs were generally considered as markers of lipidome degradation, but represented a relevant alternative to topical antibiotics, based on a biomimetic approach. In order to develop a sustainable Spirulina biorefinery scheme, different pretreatments and alternative solvents were screened to identify the best combination for the valorization of FFAs. Thus, five pre-treatments were studied including a phycocyanin extraction by-product. The following three biobased solvents were selected: ethyl acetate (EtOAc), dimethyl carbonate (DMC) and a fatty acid-based natural deep eutectic solvent (NaDES). The pigment and fatty acid profiles were established by spectroscopic and chromatographic approaches. NaDES demonstrated superior extraction capacity and selectivity compared to other biobased solvents, regardless of pretreatment. In contrast, EtOAc and DMC showed a greater diversity of FFAs, with a predominance of polyunsaturated fatty acids (PUFAs). The by-product has also been highlighted as a relevant raw material facilitating the recovery of FFAs. These results pave the way for a green biorefinery of the lipid fraction and phycobiliproteins of microalgae.

## 1. Introduction

Microalgae are sustainable and relevant resources of high-value molecules and are coveted in many sectors (human and animal nutrition, biofuel, pharmaceuticals, cosmetics) [1,2]. However, their transformation is economically difficult due to (i) the low intrinsic concentration of biomass in crops (less than 0.5%), (ii) the high-water content of the harvested biomass (often above 70%) and (iii) the variability of the intracellular composition depending on the culture conditions [3].

Due to their small size and high moisture content, microalgae can first be harvested from their growth medium and concentrated using solid–liquid separation techniques such as centrifugation. Traditionally, the biomass is then dried, pressed or freeze-dried. This intermediate stage, called pre-treatment, concerns the transformation of the biomass before extraction. The classical pretreatment processes described in the literature are divided into the following four major classes: the drying process, heat treatment, targeted fractionation/solubilization and cell disruption [3]. Biomass pretreatment aims to increase solvent diffusion by disrupting or damaging cell wall constituents. Cellular disruption can facilitate the metabolite recovery by improving the mass transfer properties of the system. Thus, the choice of pretreatment is a critical parameter in order to design a microalgae biorefinery scheme. The cell wall structure and stiffness depended on the strain of microalgae, ranging from the bare cells of *Dunaliella* to the robust cysts of *Haematococcus* [4,5], leading to intensive development for suitable pretreatment depending on the microalgae specie. The Spirulina *(Arthrospira platensis)* cell wall is composed of a succession of four layers, characteristic of the Gram-negative bacteria. The outer membrane is generally covered by a layer of polysaccharides (the glycocalyx), then, a periplasmic space separates the outer membrane and the cytoplasmic membrane, within which takes place a layer of peptidoglycans (murein) and fibers maintaining cellular rigidity [6].

Spirulina is, among all microalgae, one of the most industrially produced and extracted [7]. In 2016, around 130,000 tons of Spirulina was consumed all over the world, and this market is expected to continue to grow to reach more than 300,000 tons in the next five years, which represents revenues of almost USD 2000 Mn by 2026 [8]. *A. platensis* is mainly marketed for its high concentrations of phycobiliproteins (PBP), which represent on average between 10 and 20% of its dry weight. Once extracted and concentrated, PBP could be sold in the food, cosmetics or pharmaceutical markets, mainly for their antioxidant and fluorescent properties [9]. The increasing demand of PBP-concentrated product leads to the extraction of a growing amount of microalgal biomass, using water as the main solvent [9]. However, following this step, the remaining 80% of the cell will not be valorized. Among all the remaining metabolites, the lipidome can be still valorized, but it is usually damaged, releasing free fatty acids (FFAs).

In fact, FFAs are considered as potential new topic antibiotics, as they are known as physiological skin microbiota regulators [10]. Moreover, polyunsaturated FFAs (PUFAs) are acknowledged to be essential nutrients in cardiovascular disease prevention [11]. Spirulina is known to present a wide FFA profile, especially rich in ω-6 PUFA. The valorization of both non-polar pigment and FFAs from Spirulina is a major challenge especially considering the diversity of FFAs and the potential co-extraction with non-polar pigments (chlorophylls and carotenoids) [9]. To consider using the non-polar metabolites in cosmetics or food as an anti-ageing or microbiome regulator, it appears crucial to investigate the resulting FFA profile and the selectivity towards pigments. In fact, saturated FFAs are known to be the most potent agent towards fungi, such as *Candida albicans*, whereas ω-6 PUFAs are acknowledged to be cutaneous inflammation regulators [10]. In addition, carotenoids and chlorophylls are known as powerful anti-oxidant and anti-inflammatory metabolites [12]. Thus, an extract combining high carotenoids and FFA amounts would be a good candidate for the food or cosmetics markets.

Most FFA extraction processes described in the literature to valorize microalgae’s lipid fraction use fresh biomass and petro-sourced solvent, such as hexane or hexane/methanol. Some references reported the use of some alternative solvents to substitute hexane. Breil et al. have investigated the performances of EtOAc/MeOH and DMC/MeOH mixtures for the extraction of neutral lipids from oleaginous yeast [13]. Additionally, EtOH was explored to extract lipidic fraction from some microalgae as the wet biomass of *Pichochlorum* sp. [14], or dried biomass of *Nannochloropsis oceanica* [15]. Recently, our group explored the potential of biosourced solvent for Spirulina FFA extraction [16,17]. EtOAc and DMC were then highlighted as promising alternative solvents [16], as well as non-polar natural deep eutectic solvents (NaDES) based on fatty acids [17]. NaDES are considered as a new generation of ionic liquids, composed of a hydrogen bond acceptor (HBA) and a hydrogen bond donor (HBD). Their association forms a network of hydrogen bonds resulting in the lowering of the melting point of the solid mixtures [18,19]. Hydrophobic DES have recently emerged in the literature [20,21,22]. Those NaDES are composed of a combination of menthol or thymol with sugars, polyols or fatty acids or a combination of short chain fatty acids [21,22,23]. In a previous work, fatty acid-based NaDES were highlighted as the most promising to recover FFA from Spirulina [17]. NaDES also exhibited an increased extraction capacity allowing the decrease in biomass/solvent ratio tenfold comparing to usual solvents [17].

All this work was performed using freeze-dried biomass, which is not the most widespread pre-treatment at industrial level, considering the high economic and ecologic costs. In order to develop a sustainable Spirulina biorefinery scheme, different pretreatments and alternative solvents were screened to identify the best combination for the valorization of FFAs and non-polar pigments. Five pretreatments were then compared: cell disruption by freezing, air-, oven- or freeze-drying and by-product of selective PBP extraction. The impact on biomass integrity, dyes and lipid recovery and profile were investigated to identify the best pre-treatment for FFA and carotenoids extraction. Moreover, the extraction performance of sustainable alternative solvents (EtOAc, DMC, NaDES) was compared to heptane as reference to assess the relevance of using alternative solvents to valorize non-polar metabolites and their possible insertion in a biorefinery scheme.

## 2. Results and Discussion

### 2.1. Biomasses Characterization

*A. platensis* pretreated biomasses were first characterized as starting point. The impact of the different pretreatments on cell integrity was investigated at macroscopic and microscopic levels. Dyes and lipid content were titrated. Further, pigment diversity and the FFA profile were established using HPLC and LC-ESI-MS protocol.

Five pretreatments were screened from a PBP rich strain: cell disruption by freezing (F), air-drying (AD), oven-drying (OD), freeze-drying (FD) and by-product of selective PBP extraction (BP).

#### 2.1.1. Macroscopic Characteristics

The dry matter (DM) rate of the biomasses was measured by gravimetric measurements (Table 1). The five biomasses exhibited different DM rates, with, from the wettest to the driest: SP-BP < SP-F < SP-OD < SP-AD < SP-FD.

**Figure 1:**
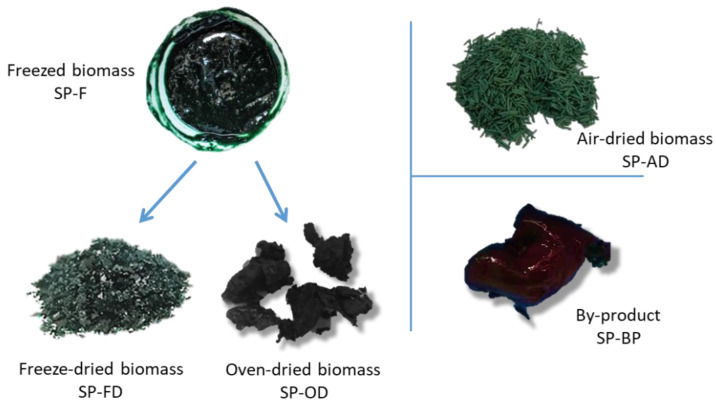
Macroscopic aspects of the different biomasses studied.

This observation was consistent with the macroscopic aspect (Figure 1). The two wettest biomasses, SP-BP and SP-F (17.2% and 25.8% of DM, respectively) had the form of viscous pastes while the dried biomasses take the form of powder (SP-FD; 99.3% DM) or brittle flakes (SP-AD; 93.1% DM). The oven-dried form had an intermediate dry matter content (77.6%). As it dried, the paste formed flexible aggregates (dry on the surface but soft inside). As the pretreatment could modify the cellular structure of the biomasses, a microscopic observation was carried out.

#### 2.1.2. Cell Integrity

*A. platensis* is morphologically characterized by long, unbranched, spirally coiled filaments. The length and curl might vary depending on growing conditions.

**Figure 2:**
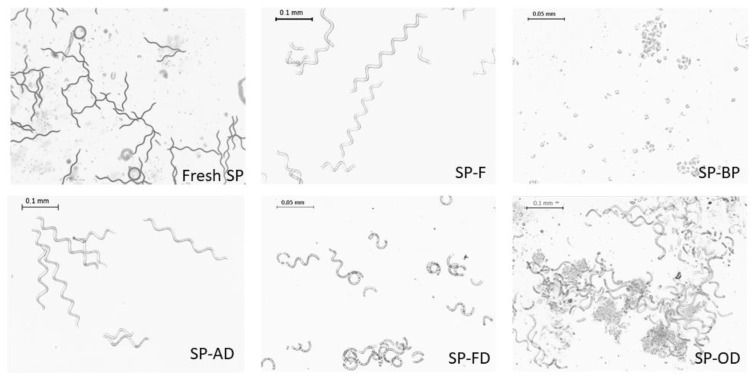
Microscopic photographs of pretreated Spirulina’s biomasses. Fresh SP (biomass just after harvest); F (Frozen biomass); OD (Oven-dried); FD (Freeze-dried); D (Air-dried); and BP (By-product of PBP extraction).

The freezing (SP-F) and air-dried (SP-AD) processes led to the least impact on the cell wall integrity. As shown in Figure 2, typical long spiral structures, about 250 µm long, were observed as the dominant form.

The freeze-drying process also partially preserved the structure of the cells, since long filaments were still visible even if shorter spirals were apparent. A majority of debris was observed for the biomass dried in the oven (SP-OD). In contrast, the prior extraction of phycocyanin (SP-BP) showed the highest cellular impact with the appearance of predominant cellular debris. Cells were individualized, cubic in shape, and measured between 5 to 7.5 µm in length.

The impact on the integrity of the pretreatment was therefore an important factor to take into account, with cellular structures ranging from a very preserved state to a totally degraded state (SP-F to SP-BP). After these organoleptic characterizations, the cell content and, in particular, dye and lipid contents were explored.

#### 2.1.3. Dye Content and Profile

Considering pigment access (Table 1), differences were noticed between pretreated biomasses. Considering *A. platensis*, phycocyanin remained the major compound found in the biomass with rates ranging from 82 to 120 mg/g DM, even for the by-product already pre-extracted. Chlorophylls and carotenoids were found at lower levels (from 2.7 to 13.3 mg/g DM and from 1.3 to 4.8 mg/g DM, respectively). The drying processes (oven, air, freeze-drying) seemed to reduce access to the pigments compared to the freezing process, which is probably related to a degradation of these metabolites known for their low stability [24].

**Figure 3:**
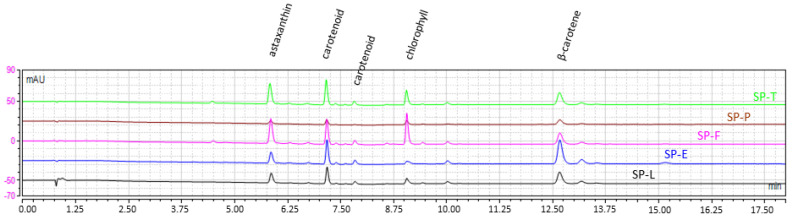
HPLC chromatogram at 450 nm of Spirulina’s biomasses chloroformic extracts.

Regarding carotenoids at the qualitative level, astaxanthin and β-carotene were identified in all biomasses (Figure 3).

They were present mainly in wet biomasses (SP-F and SP-BP) and in a lesser content in dried biomasses (SP-AD, SP-FD and SP-OD). The chlorophylls and pheophytins identified were mainly of type *a*, according to their UV spectra. Their concentrations seemed to decrease with thermal pretreatments.

#### 2.1.4. Lipid Content

To understand the impact of pretreatment on cell degradation, access to lipid content was studied. The results of the total lipid assay appeared in Table 1. The air-dried form (SP-AD) showed the lowest total lipid contents with 13.4 mg eq castor oil/g DM. The SP-FD, SP-F and SP-OD biomasses had relatively similar rates ranging from 20.7 to 26.5 mg eq castor oil/g DM. The highest level was observed for SP-BP with 37.3 mg eq castor oil/g DM. By comparing these results with the microscopic structure of each of the biomasses, the states that present the most damaged cells were also those that had the highest contents of extractable lipids (SP-BP and SP-OD), while the driest biomasses had the lowest lipid levels (i.e., SP-AD and SP-FD). A dry state therefore did not seem conducive to the diffusion of the reagent; the latter may be too non-polar to rehydrate the biomass. Conversely, wet biomass, whose pretreatment preserved the cell structure, such as freezing, did not seem favorable either.

In addition to access to extractable lipids, it was essential to analyze the FFA profile of the different biomasses, in order to select the best biomass.

#### 2.1.5. FFA Profile

**Figure 4:**
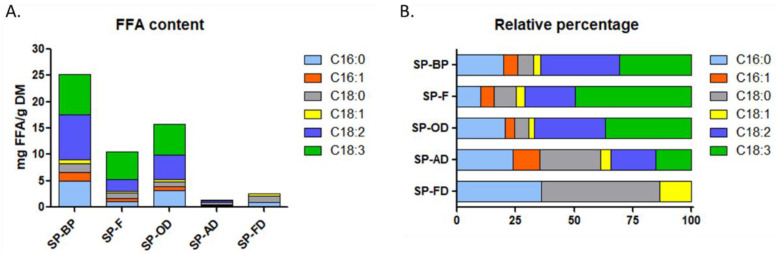
FFA content of *A. platensis* expressed as mg/g DM ((**A**)-left) and in relative percentage ((**B**)-right).

Chloroformic fractions from the SPV assay were analyzed by LC-ESI-MS in order to obtain the FFA profile summarized in Figure 4 and Table S1.

FFAs were barely detectable in dried SP-FD and SP-AD biomasses, consistent with the results obtained for extractable lipids. Overall, SP-BP had the highest FFA levels (25 mg/g DM), followed by the oven-dried (SP-OD) biomass (15 mg/g DM) and the frozen one (10 mg/g DM). This result was not surprising considering the degradation of the cells. Interestingly, even though the impact of freezing (SP-F) and drying (SP-OD) processes did not appear to be significantly different upon microscopic analysis, the impact on FFA access or generation was important. In fact, the FFA content was almost tenfold higher in SP-F than in SP-AD. The hydration rate of the biomass thus seemed to play a key role in lipids preservation and in access to FFAs in particular.

Concerning the qualitative profile in FFAs, low levels of palmitic acid have been observed (10–35%), whereas in the literature the average level described was 40% [25]. Biomasses with low dry matter (SP-F and SP-BP), but also SP-OD, contain fairly high levels of ω-6 type PUFAs, particularly linoleic (from 2 to 8 mg/g DM) and γ-linolenic acids (5 to 7 mg/g DM), in accordance with the literature [25]. Thus, considering the diversity of FFAs, SP-F, SP-OD and SP-BP have similar relative profiles, dominated by PUFAs, in particular ω-6. On the contrary, the relative amount of free PUFAs was considerably decreased in the SP-FD and SP-AD biomasses. As expected, the drying process impacted the relative free PUFA percentage from 71% in SP-F to 34% in SP-AD. Esquivel et al. showed that air-drying (in a convection oven at 30 °C) on *Phaeodactylum tricornotum* caused a loss of 70% of the total lipids immediately after the dying process, while this was not the case when freezing. The reason for the high percentages of lipids lost initially with the drying technique is possibly due to the fact that microalgae store at least part of their lipids as oil droplets, which are more volatile than other types of lipids even at low temperatures [26]. Moreover, Oliveira et al. noted an effect of air drying on the degree of oxidation on Spirulina. The degree of FFA unsaturation decreased slightly at higher temperature for all samples with damaged cell walls, this state favoring the oxidation of unsaturated bonds. [27]. Here, the lower amount of FFAs in SP-AD or FD was more likely linked to lipidome preservation. Indeed, since FFA are generally used as markers of lipidome degradation, it was consistent that pretreatments that preserve the most cells generate the least amount of lipidome degradation, i.e. the lowest amount of FFA. This hypothesis was also validated by the absence of PUFA in the extracts produced with the freeze-dried biomass. Paradoxically, the by-product and the frozen biomass appeared as the most relevant pretreatment for FFA extraction, highlighting the probable high level of lipidome degradation.

### 2.2. Extraction

Three alternative solvents were selected from the previous data of our group [10,11]: two biosourced solvents, EtOAc and DMC, and one ternary NaDES C9/C10/C12 (3:2:1, mol/mol). Heptane was selected as an industrial reference solvent. An ultrasonic assisted extraction (UAE) was performed using two biomass/solvent ratios depending on the solvent nature. We had previously demonstrated that a ratio of 1/20 was optimal using EtOAc, DMC and heptane [16] and that a ratio of 1/6 was optimized using this NaDES [17]. All titrations were then referred to the DM of the engaged biomass, allowing then the comparison of dry and humid biomasses as well as regular solvent and NaDES, which were non-volatile.

#### 2.2.1. Cell Integrity

**Figure 5:**
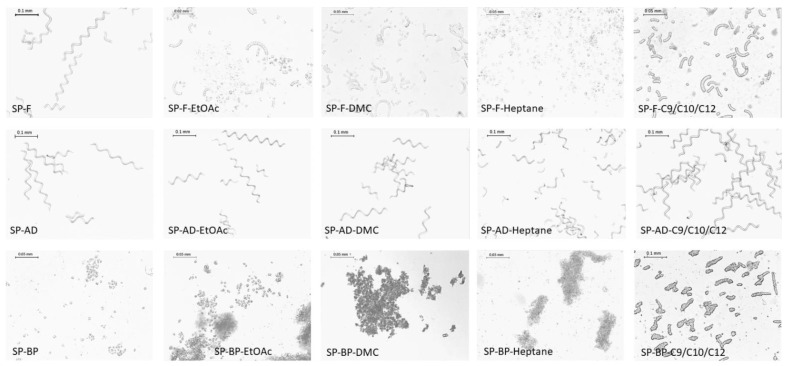
Photographs of *A. platensis* biomasses after extraction with green and reference solvents. F (Frozen biomass); OD (Oven-dried); FD (Freeze-dried); AD (Air-dried); and BP (By-product of phycobiliprotein extraction). EtOAc, ethylacetate; DMC, dimethylcarbonate; NaDES C9/C10/C12 (3:2:1: mol/mol).

After extraction, all the biomasses were observed under a microscope in order to identify possible changes in their cellular integrity after ultrasonic treatment (Figure 5).

Frozen Spirulina cells (SP-F) were severely damaged after extraction. Microscopic observation showed a dominance of broken spirals after extraction with EtOAc, DMC or C9/C10/C12, whereas the cells were completely individualized in the presence of heptane (4 to 6 µm in length). If, for the SP-AD biomass, the extraction did not seem to have an impact on the cell structure whatever the solvent, SP-BP cells were more damaged after the extraction process. Hydrated cells formed agglomerates in the presence of non-polar solvent. In the case of DMC and heptane, the cells totally exploded, and the cellular contents then formed a cloud of debris in the medium (Figure 5).

The air-dried biomass exhibited the highest stability again even after UAE, and long spiral cells were still observed at all conditions.

#### 2.2.2. Pigment and Lipid Content

Only hydrophobic pigments (chlorophylls and carotenoids) and extractable lipids were quantified by a UV-visible spectrophotometric assay, as PBP were too polar to be extracted by these non-polar solvents (Table 2) [28].

The extraction yield with organic solvents remained low: 1% on dried and freeze-dried biomass, 2–8% on fresh frozen biomass and 7–16% on by-product. It is important to note that extraction yields were also not sufficient to carry out assay triplicates for SP-OD and SP-FD with EtOAc or DMC.

SP-OD and SP-BP exhibited the best total lipid levels, surely favored by destabilized cell walls, while SP-F and SP-BP had the best pigment levels. Contradicting results were reported by Ehimen et al. who observed a lower lipid extraction efficiency from wet biomass than from dry biomass, considering lipids extraction for transesterification [29].

The lipid extraction performances of DMC and EtOAc followed the trend observed on non-extracted biomass, i.e., SP-BP > SP-OD > SP-F > SP-AD > SP-FD. They were therefore as effective on wet biomass as on dry biomass, whereas heptane was found to be more effective on dry biomass. EtOAc seemed to be more selective for pigments (in particular, chlorophylls) than the other solvents tested, even if access to pigments was generally not favored.

Considering the non-polar NaDES, the total lipid content was not realized as NaDES components were known to disturb the SVR reaction [17]. We could also note that the selectivity of NaDES towards FFA was higher than that of biosourced solvents, especially EtOAc.In fact, for each pretreatment, the pigment amounts in the NaDES extract were lower than in EtOAc.

#### 2.2.3. FFA Profile

**Figure 6:**
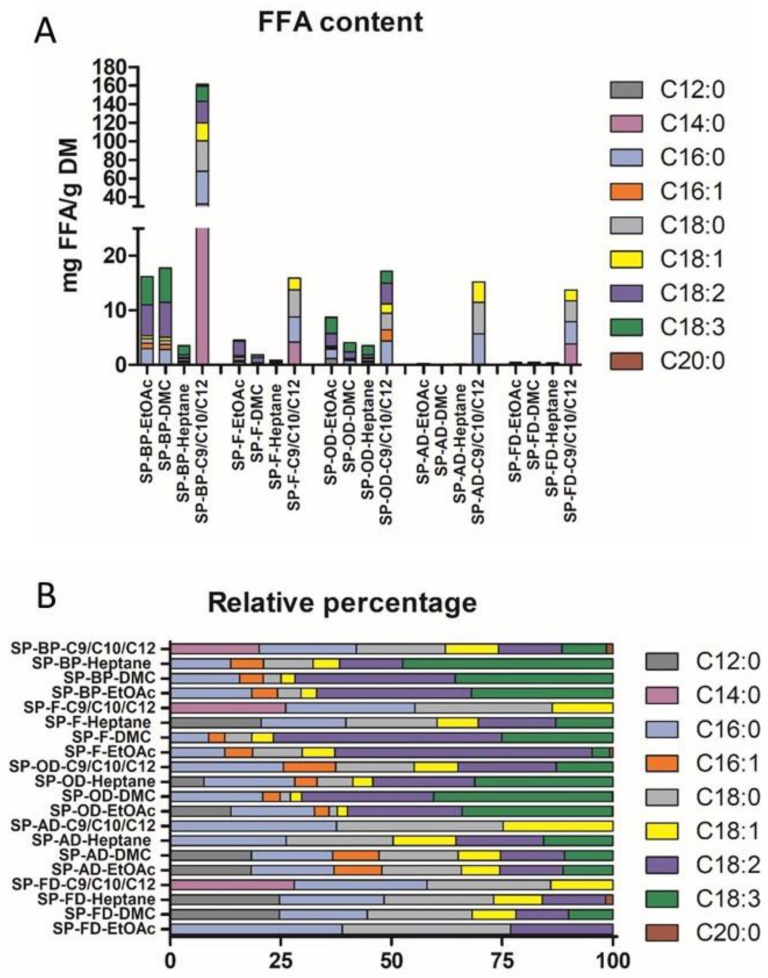
FFA content of *A. platensis* expressed as mg/g DM ((**A**)-top) and in relative percentage ((**B**)-bottom).

Data obtained by LC-ESI-MS have been summarized in Figure 6 and Table S2.

All solvents combined, the extracts obtained from the by-product have a much higher FFA content than other biomasses with 3.51 mg/g DM for SP-BP-Heptane, and between 16 and 17 mg/g DM for, respectively, SP-BP-EtOAc and -DMC, and reaching up to 161.5 mg/g DM for the NaDES extract.

Lower extraction efficiency was observed with dried biomasses, except for NaDES where no significant difference was found between frozen (SP-F) and dried biomasses (SP-AD, SP-OD and SP-FD). As the structure is less damaged, the cell wall probably acted as a barrier that decreased solvent penetration and mass transfer. Again, pretreatments could be classed according to their performances (from the best to the worst): SP-BP > SP-OD > SP-F > SP-AD ≈ SP-FD.

Further, drying processes (OD and AD) limited the FFA extraction. The study conducted by Zepka et al. reported an increase in lipid yield from *Aphanothece microscopic* by drying at 60 °C instead of 40 °C and showed by this study that drying temperature had a significant impact on the lipid yield [30]. However, these results contradicted the report by Widjaja et al., as they had observed significant reduction in lipid yield in *Chlorella vulgaris* when drying at higher temperatures [31]. The drying temperature during lipid extraction from algal biomass was found to affect not only the lipid composition but also lipid content. The oxidation of fatty acid upon exposure to high temperatures has been reported and unsaturated fatty acid, especially polyunsaturated free fatty acid (PUFA), was more susceptible to oxidation than saturated fatty acid. The inconsistency of temperature effect on lipid recovery may be due to the differences in microalgae species and associated fatty acid profiles, such as the level of unsaturation and amount of free fatty acids.

For all types of biomasses combined, the ternary NaDES C9/C10/C12 showed better selectivity. SP-BP-NaDES had a FFA level tenfold higher than organic solvents and extracted up to 161 mg FFA/g DM. NaDES seemed to be more selective for FFA than conventional solvents, but the extract obtained was enriched in saturated FFA while the FFA profile of biosourced solvents reflects the composition of the initial biomass, which was richer in PUFA and MUFA. Otherwise, the observed diversity was in accordance with the FFA profile of the initial biomasses.

**Figure 7:**
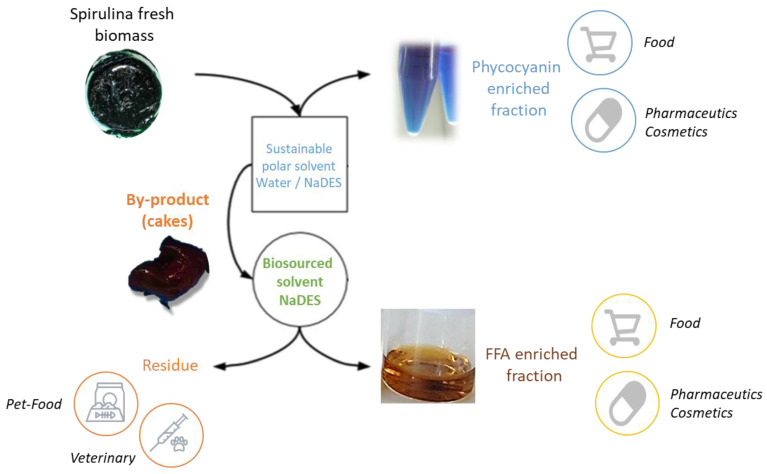
Biorefinery scheme proposed for Spirulina valorization with possible markets.

All these results led to the following conclusions: in order to valorize the by-product of PBP water extraction, FFA appeared as a real opportunity of value-added compounds production for the cosmetics, food and pharmaceutical markets. This work was thus paving the way for Spirulina biorefinery. A proposed biorefinery scheme is described in Figure 7.

Starting from fresh Spirulina biomass, a first step of polar extraction was proposed in order to extract PBB for the food, pharmaceutical or cosmetics markets. A sustainable solvent might be used as the widespread water [28]. Recently, polar NaDES were also highlighted as relevant solvents for PBP extraction from fresh Spirulina biomass [32]. NaDES, especially those based on glucose, acted as a stability enhancer beside their good extraction performances. Moreover, their use allowed for water consumption reduction, in accordance with eco-extraction guidelines.

Then, the resulting by-product could be valorized using a sustainable non-polar solvent, targeting FFA for cosmetics, pharmaceutics or food. Regarding the durability of the screened solvent, EtOAc and DMC exhibited lower recovery and higher solvent consumption than NaDES. Indeed, the biomass/solvent ratio was three times higher using a conventional biobased solvent, leading to a dramatic increase in the cost of extraction. In fact, considering only the cost of each solvent, the NADES (52.4 EUR/L) appeared as the most expensive option compared to EtOAc (25.3 EUR/L) and DMC (37.8 EUR/L) (prices from Fisher Scientific in September 2022). However, taking into account the consumption of solvent requested to extract 1 kg of biomass, DMC was then highlighted as the less economically relevant option with a cost of 756.0 EUR per kg biomass extracted, not so far from EtOAc (505.4 EUR/kg biomass), but far away from NaDES with a cost divided almost by two (400.4 EUR/kg biomass).

On the other hand, these organic solvents could be evaporated and recycled to improve the sustainability score. Considering NaDES, as a non-volatile and biocompatible solvent, it could remain in the final product, with its composition being compliant with cosmetic, food or pharmaceutical regulations. In this case, the solvent removal step would be totally avoided, resulting in a significant improvement in productivity, combined with less solvent consumption. The fatty acid-based NaDES, although the selectivity and recovery were very high, selectively extracted saturated FFAs with poor recovery of PUFAs. The composition of these NaDES must be customized to modulate their selectivity by modifying the thermodynamic interaction profile. Indeed, in NaDES based on pure fatty acids, the interactions were dominated by Van der Waals-type bonds, which are not the most relevant for targeting PUFAs. Inspired by EtOAc and DMC, a fatty ester or carbonate associated with fatty acids would be an interesting approach to explore.

The last point to take into consideration was the safety of these solvents for the people working on the extraction plan. NaDES in this case offer many more guaranties as a non-volatile mixture.

Finally, the final residue, still rich in chlorophyll and carotenoids, could be valorized in pet food or for veterinary used, allowing then the total valorization of the biomass.

## 3. Materials and Methods

### 3.1. Raw Material

Spirulina (*Arthrospira platensis*) biomasses were kindly provided by Aqua Eco Culture (Lamballe, France). Five different states of biomass were investigated as mentioned. One part of the fresh biomass was frozen just after harvesting at −20 °C (SP-F). The second part was air-dried gently using the internal Aqua Eco Culture process (SP-AD). Once frozen, a part of the biomass was dried at 45 °C overnight in a convection oven (UN30, Memmert) to obtain the oven-dried biomass (SP-OD) and another part was freeze-dried for 24 h (Leybold-Heraeus) (SP-FD) as described in Figure 1. By-product was obtained after the extraction of phycobiliprotein using the internal Aqua Eco Culture process. The dry matter (DM) content of each kind of biomass was resumed in Table 1. All the biomasses were stocked at −20 °C.

### 3.2. Chemicals

Ethyl acetate (EtOAc), methanol (MeOH), acetonitrile (ACN), chloroform (CHCl_3_), sulfuric acid 96% (H_2_SO_4_) and propan-2-ol were purchased from Carlo Erba (Val de Reuil, France). Ammonium formate and the following standards were purchased from Sigma Aldrich (Saint-Quentin Fallavier, France): dodecanoic acid, myristic acid, palmitic acid, palmitoleic acid, stearic acid, oleic acid, linoleic acid, γ-linolenic acid, arachidic acid, arachidonic acid, nervonic acid, eicosapentaenoic acid and eicosatrienoic acid. Dimethyl carbonate (DMC) and lauric acid 99% were purchased from Acros Organics (Geel, Belgium). Formic acid was purchased from Fisher Scientific SAS (Illkirch, France). Nonanoic acid 98% and decanoic acid 99% were purchased from Alfa Aesar (Haverhill, MA, USA). Water was purified using a Milli-Q system (Millipore Corporation, Bedford, MA, USA). Phosphoric acid 85–90% (H_3_PO_4_) and formic acid were purchased from Fisher Scientific SAS (Illkirch, France). Vanillin was purchased from Extrasynthese (Genay, France).

### 3.3. NaDES Preparation

The ternary NADES C9/C10/C12 at a molar ratio 3:2:1 was prepared by mixing components together. The mixture was heated at 50 °C and stirred until a colorless liquid was obtained.

### 3.4. Extraction Process

Biomass was extracted with EtOAc, DMC or heptane using ultrasonic assisted extraction (UAE) for 30 min with biomass/solvent ratio (1/20, *w*/*w*). The resulting extracts were centrifuged at 16,200× *g* (Rotanta 460 R, Hettich, Kirchlengern, Germany) for 20 min. Supernatant was collected and solid residue was extracted two more times (final biomass/solvent ratio 1/60). After the last cycle, the resulting supernatants were pooled, concentrated under vacuum and stocked at 6 °C. Each extraction was performed in triplicate [16].

For NaDES extraction, the protocol followed was the same as that described above with a final biomass/solvent ratio of 1/6 [17].

### 3.5. Analytical Protocols

All extracts and the initial biomass were analyzed in terms of phycobiliprotein, chlorophyll and carotenoid content, lipid content and FFA and dyes profiles. Spirulina extracts were named “SP-X-Y” according to the pre-treatment and the solvent used. For example, the resulting frozen Spirulina extract obtained with EtOAc was named “SP-F-EtOAc”.

#### 3.5.1. Microscopy

The cells before and after extraction were observed under trinocular microscope LEICA L3000 and photographed using a digital camera LEICA EC3 (Leica Camera AG, Wetzlar, Germany). Photos were edited with the LEICA associated software LAS EZ version 1.6.0. The magnification was ×200 (frozen, freeze-dried, air-dried, oven-dried and by product) or ×100 (fresh biomass).

#### 3.5.2. Dye Content

The initial biomasses were characterized in terms of global dye content. Biomasses were extracted by water under magnetic agitation for 2 h with biomass dry matter/solvent ratio (1/25, *w*/*w*). An amount of 1 mL of each sample was centrifuged for 10 min with a Mini-Centrifuge (Fisher Scientific SAS, Illkirch, France). The supernatant was then recovered and distributed in 96 well microplates. The absorbance was measured at 620, 652 nm for C-phycocyanin and 565 nm using a Multiskan GO plate reader (ThermoFisher Scientific, Villebon Coutaboeuf, France) with SkanIt RE software. The resulting solid residues were then dissolved in 1 mL of MeOH and the absorbance was measured at 450, 645 and 666 nm. The mass concentration of B-phycocyanin was obtained thanks to the equation described by Benett et al. [33] and the calculations for chlorophylls and carotenoids were established thanks to the equation described by Henriques et al. [34]. Error was expressed as standard deviation.

#### 3.5.3. Dye Profile

The qualitative chlorophylls/carotenoids profile was determined using high-performance liquid chromatography (HPLC) based on [35]. The samples from the titration assay were then filtered through a syringe filter with a porosity of 0.45 µm and transferred into HPLC vials. The samples were analyzed on a Dionex U3000RS HPLC chain equipped with a diode array (Thermo Fisher Scientific SAS). An amount of 5 µL of each sample was then injected into a column (Accucore aQ 150 mm × 3 mm × 2.6 µm) accompanied by a precolumn (13 mm × 0.3 mm) (Thermo Fisher Scientific SAS). The flow rate was set at 0.8 mL/min and the column temperature was maintained at 30 °C. The mobile phases were as follows: (A) MeOH/ammonium acetate 0.1 M (aq) (7:3, *v*/*v*) and (B) MeOH 100%. The gradient was set as follows: initial solvent B content was 25%, raised to 50% in 0.64 min, then 100% in 6.6 min and maintained for 17 min.

#### 3.5.4. Lipid Content

The total lipid content of initial biomasses and extracts was evaluated using the sulpho-phospho-vanillin protocol. The rapid total lipid assay was adapted from [36]. Samples and reference castor oil were dissolved in CHCl_3_/methanol (2:1, *v*/*v*) at 10 mg dry biomass/mL for initial biomasses and at 1 mg/mL for extracts. Samples were then evaporated at 90 °C. Solid residue was solubilized in 0.1 mL of H_2_SO_4_ 96% and then heated for 10 min at 90 °C. Samples were cooled for 5 min in ice and then mixed with a phosphovanillic reagent (PVR) (1.2 mg of vanillin in a mixture of 8 mL of 85% H_3_PO_4_ and 2 mL of water). Absorbance at 530 nm was then measured using a Multiskan GO plate reader (ThermoFisher Scientific, Villebon Coutaboeuf, France) with SkanIt RE software. Results were expressed as mg of equivalent of castor oil per g of dry matter; error was expressed as standard deviation.

#### 3.5.5. FFA Profile

The FFA content of biomass was determined using liquid chromatography-mass spectroscopy (LC-ESI-MS). LC-ESI-MS analyses were performed on an Acquity H-Class with a SQD detector (Waters, Saint Quentin en Yvelines, France). The system was fitted with a BEH C18 (50 mm × 2.1 mm; 1.7 µm particle size). The column oven was set at 60 °C.

The mobile phases were (A) 0.01% formic acid (aq) containing 0.2 mM ammonium formate and (B) 50% isopropanol in acetonitrile containing 0.01% formic acid (aq) and 0.2 mM ammonium formate. The flow rate was 0.24 mL min^−1^ and gradient was set as follows: initial solvent B content was 50%, raised to 98% in 16 min and maintained for 4 min. The column was then re-equilibrated to initial conditions. ESI in negative mode was performed with cone voltage set at 45 V and capillary voltage at 3.5 kV. NaDES extracts required a pretreatment prior to LC-ESI-MS analyses. Solid-phase extraction was performed using C18-silica cartridges (HyperSep 1 g, 40–60 µm, Fischer Scientific, Waltham, MA, USA) with an elution gradient of different mixtures of water and MeOH ranging from 50 to 100% of MeOH. The 100% methanolic fractions were analyzed. Samples were prepared in MeOH. Nervonic acid (C20:4) at 10 µg/mL was added as internal standard. All FFAs were identified by comparison of retention time and mass spectra using commercial standards (Sigma Fatty acids Saturated and unsaturated kit). FFA titration was performed using a calibration curve of each standard ranging from 2.5 µg/mL to 12.5 µg/mL (1 µL injection) and using nervonic acid as internal standard. Linear regression and calculation were performed of SIR chromatogram using Targetlynx (Waters). The *m*/*z* and retention time of all standards were summarized in Table S3.

For initial biomasses, 10 mg of biomass was extracted by 1 mL of CHCl_3_/methanol (2:1, *v*/*v*) and then evaporated to dryness prior to analysis. Conventional extracts and chloroformic fractions were concentrated at 250 µg/mL, whereas the methanolic fractions from NaDES extracts were concentrated at 100 µg/mL. Then, 1 µL of the samples were injected in the system.

### 3.6. Statistical Analysis

The results were analyzed by GraphPad Prism version 5 (GraphPad Software, La Jolla, CA, USA). The statistical significance was evaluated by the one-way ANOVA Kruskall–Wallis test followed by the Dunn’s comparison test. Differences were considered to be significant at *p* < 0.05.

## 4. Conclusions

The pretreatment of Spirulina biomasses was, as expected, a key parameter for the valorization of FFAs. On the one hand, the air-drying process right after harvest was highlighted as the most conservative, as cell integrity was preserved even after ultrasonic treatment. This has opened new perspectives for the preservation of biomass after harvest. On the other hand, the by-product of PBP has been put forward as a relevant raw material facilitating the valorization of FFAs. Indeed, the drastic treatment applied to the Spirulina biomasses during the extraction of the PBP led to the degradation of the lipidome, which released a large quantity of FFA. This problem was considered in our case as an advantage since the amount of target compounds was considerably increased.

NaDES C9/C10/C12 based on fatty acids have demonstrated superior extraction capacity and increased selectivity compared to other biobased solvents, regardless of the pretreatment. In contrast, EtOAc and DMC showed a greater diversity of FFAs, with a predominance of polyunsaturated fatty acids (PUFAs). The modulation of the composition of NaDES is currently being studied in our group using both an in silico and experimental approach to improve the selectivity of NaDES towards PUFAs.

These results pave the way for a green biorefinery of the lipid fraction and phycobiliproteins of microalgae.

## Figures and Tables

**Table 1:** Biomasses’ characteristics (dry matter, pigment and lipid content). Data represent the mean ± SD. DM = dry matter.

Microalgae	Pretreatment (Code)	DM (%)	Phycocyanin (mg/g DM) *n* = 3	Chlorophylls (mg/g DM) *n* = 3	Carotenoids (mg/g DM) *n* = 3	Total Lipid (mg eq Castor Oil/g DM) *n* = 3
*A. platensis*	By-product SP-BP	17.2	84.8 ± 0.4	4.4 ± 0.1	1.6 ± 0.1	37.3 ± 2.7
	Frozen SP-F	25.8	120.9 ± 0.9	13.3 ± 0.5	4.8 ± 0.2	22.4 ± 2.0
	Oven-dried SP-OD	77.6	94.3 ± 1.3	2.7 ± 0.1	1.5 ± 0.0	26.5 ± 3.2
	Air-dried SP-AD	93.1	82.2 ± 2.9	4.3 ± 0.1	1.3 ± 0.0	13.4 ± 0.5
	Freeze-dried SP-FD	99.3	98.9 ± 0.7	5.9 ± 0.6	1.7 ± 0.2	20.7 ± 1.7

**Table 2:** Extracts’ characteristics (pigment and lipid content). Data represent the mean ± SD. DM = dry matter.

Pretreatment	Solvent	Chlorophylls (mg/g DM) *n* = 3	Carotenoids (mg/g DM) *n* = 3	Total Lipid (mg eq Castor Oil/g DM) *n* = 3
SP-BP	EtOAc	19.0 ± 0.52	6.21 ± 0.16	308.4 ± 38.6
	DMC	8.86 ± 0.52	4.76 ± 0.24	414.5 ± 35.2
	Heptane	0.02 ± 0.00	0.00 ± 0.00	131.2 ± 25.6
	C9/C10/C12	5.75 ± 0.09	0.15 ± 0.06	/
SP-F	EtOAc	1.25 ± 0.00	1.25 ± 0.00	256.8 ± 23.3
	DMC	0.19 ± 0.00	0.14 ± 0.00	324.6 ± 33.2
	Heptane	0.02 ± 0.00	0.00 ± 0.00	188.8 ± 9.8
	C9/C10/C12	0.27 ± 0.09	0.03 ± 0.02	/
SP-OD	EtOAc	0.45	0.40	323.3 ± 22.2
	DMC	0.24 ± 0.00	0.17 ± 0.02	417.4 ± 31.37
	Heptane	0.03 ± 0.00	0.07 ± 0.00	242.9 ± 34.4
	C9/C10/C12	2.01 ± 0.12	1.05 ± 0.08	/
SP-AD	EtOAc	0.50 ± 0.14	0.26 ± 0.01	255.0 ± 41.2
	DMC	0.02 ± 0.00	0.01 ± 0.00	225.0 ± 12.0
	Heptane	0.01 ± 0.00	0.02 ± 0.00	217.6 ± 35.5
	C9/C10/C12	0.16 ± 0.03	0.01 ± 0.00	/
SP-FD	EtOAc	1.42	0.06	303.5 ± 9.8
	DMC	0.58	0.31	173.9 ± 12.7
	Heptane	0.04 ± 0.00	0.2 ± 0.00	264.9 ± 21.3
	C9/C10/C12	1.02 ± 0.15	0.73 ± 0.09	/

## Data Availability

Raw datasets generated during this study are available from the corresponding authors upon reasonable request.

## Supplementary Materials

The following supporting information can be downloaded at: https://www.mdpi.com/article/10.3390/md20100600/s1, Table S1: Amount of FFA in biomasses in µg/g DM; Table S2: Average amount of FFA in extracts in mg/g DM; Table S3: Retention time and m/z (ESI-) of free fatty acids.
