# Supplementary material for: Alternative Solvents for the Biorefinery of Spirulina: Impact of Pretreatment on Free Fatty Acids with High Added Value
Source: Mar Drugs. 2022 Sep 25;20(10):600. doi: 10.3390/md20100600 (PMC9605531; doi:10.3390/md20100600)
Supplement: Supplementary file 1 [file marinedrugs-20-00600-s001.zip › marinedrugs-1914664-supplementary.pdf]

Alternative Solvents for the Biorefinery of Spirulina: Impact of Pretreatment on Free Fatty Acids with High Added Value

Laura Wils [1], Mervé Yagmur [1], Myriam Phelippe [2], Bénédicte Montigny [3], Barbara Clément-Larosière [2], Johan Jacquemin [4] and Leslie Boudesocque-Delaye [1,*]

[1] EA 7502 SIMBA, Université de Tours, Faculté de Pharmacie, 31 avenue Monge 37200 Tours, France
[2] Aqua Eco Culture, 7 rue d'Armor Maroué, 22400 Lamballe, France
[3] EA 6299 PCM2E, Université de Tours, Faculté des Sciences et Techniques, Bât J, Avenue Monge, 37200 Tours, France
[4] MSN Department Mohammed VI Polytechnic University, Lot 660, Hay Moulay Rachid, Ben Guerir 43150, Morocco
* Correspondence: leslie.boudesocque@univ-tours.fr; +33(0)2.47.36.71.75

Supplementary material

Table S1 Amount of FFA in biomasses in μg/g DM, *n.d.* not detected

Pretreatment	*C 12:0*	*C14:0*	*C 16:0*	*C18:0*	*C16:1*	*C18:1*	*C18:2*	*C18:3*
By-product SP-BP	*n.d.*	*n.d*	504.0 ± 7.8	171.0 ± 51.1	153.0 ± 7.8	72.0 ± 15.6	855.0 ± 68.0	796.5 ± 38.1
Frozen SP-F	*n.d*	*n.d*	105.0 ± 7.5	100.0 ± 10.7	60.0 ± 0.0	37.5 ± 0.0	227.5 ± 15.6	517.5 ± 27.0
Oven-dried SP-OD	*n.d*	*n.d*	322.8 ± 27.1	95.3 ± 7.5	62.8 ± 9.9	41.2 ± 7.5	476.7 ± 22.8	578.5 ± 39.3
Air-dried SP-AD	*n.d*	*n.d*	33.3 ± 4.2	36.0 ± 3.5	16.0 ± 0.0	6.0 ± 2.8	26.7 ± 1.1	21.3 ± 1.2
Freeze-dried SP-FD	*n.d*	*n.d*	90.7 ± 13.0	127.5 ± 8.5	*n.d.*	34.0 ± 0.0	*n.d.*	*n.d.*

Table S2 Average amount of FFA in extracts in mg/g DM

Pretreatment	*Solvent*	*C12:0*	*C14:0*	*C 16:0*	*C18:0*	*C16:1*	*C18:1*	*C18:2*	*C18:3*
	EtOAc	*n.d*	*n.d*	2.90	0.85	0.94	0.58	5.66	5.17
By-product	DMC	*n.d*	*n.d*	2.83	0.71	0.94	0.55	6.43	6.35
SP-BP	Heptane	*n.d*	*n.d*	0.45	0.40	0.261	0.21	0.50	1.67
	C9/C10/C12	*n.d*	32.46	35.5	32.44	*n.d*	19.31	23.18	16.22
	EtOAc	*n.d*	*n.d*	0.56	0.51	0.28	0.34	2.63	0.18
Frozen	DMC	*n.d*	*n.d*	0.16	0.11	0.07	0.09	0.97	0.47
SP-F	Heptane	0.18	*n.d*	0.16	0.18	*n.d*	0.08	0.15	0.11
	C9/C10/C12		4.16	4.65	4.95	*n.d*	2.18	*n.d*	*n.d*
	EtOAc	1.20	*n.d*	1.66	0.16	0.28	0.20	2.26	2.97
Oven-dried	DMC	*n.d*	*n.d*	0.85	0.09	0.16	0.10	1.21	1.64
SP-OD	Heptane	*n.d*	*n.d*	0.48	0.40	0.26	0.21	0.50	1.67
	C9/C10/C12	*n.d*	*n.d*	4.41	3.05	2.03	1.70	3.82	2.20
	EtOAc	0.05	*n.d*	0.06	0.05	0.03	0.03	0.04	0.03
Air-dried	DMC	0.02	*n.d*	0.02	0.01	0.01	0.01	0.01	0.01
SP-AD	Heptane	*n.d*	*n.d*	0.05	0.05	*n.d*	0.03	0.04	0.03
	C9/C10/C12	*n.d*	*n.d*	5.73	5.73	*n.d*	3.78	*n.d*	*n.d*
	EtOAc	*n.d*	*n.d*	0.18	0.17	*n.d*	*n.d*	0.10	*n.d*
Freeze-dried	DMC	0.11	*n.d*	0.09	0.11	*n.d*	0.04	0.05	0.05
SP-FD	Heptane	0.10	*n.d*	0.09	0.10	*n.d*	0.04	0.06	*n.d*
	C9/C10/C12	*n.d*	3.83	4.11	3.84	*n.d*	1.92	*n.d*	*n.d*

Table S3: Retention time and m/z (ESI-) of free fatty acids

Fatty acids		m/z	RT (min)
**C12:0**	lauric acid	199.3	4.31
**C14:0**	myristic acid	227.4	6.40
**C16:0**	palmitic acid	255.4	8.43
**C16:1**	palmitoleic acid	253.4	6.81
**C18:0**	stearic acid	283.5	10.27
**C18:1. ω-9**	oleic acid	281.4	8.74
**C18:2. ω-6**	linoleic acid	279.4	7.38
**C18:3. ω-6**	ϒ-linolenic acid	277.25	6.16
**C24:1. ω-9**	nervonic acid	365.6	13.16
